# GSTM3 A/B Polymorphism and Risk for Head and Neck Cancer: A Meta-Analysis

**DOI:** 10.1371/journal.pone.0083851

**Published:** 2014-01-08

**Authors:** Yu Xu, Jun Wang, Weiguo Dong

**Affiliations:** 1 Department of Otolaryngology, Renmin Hospital of Wuhan University, Wuhan, Hubei Province, China; 2 Department of Gastroenterology, Renmin Hospital of Wuhan University, Wuhan, Hubei Province, China; Sapporo Medical University, Japan

## Abstract

**Background:**

Glutathione S-transferase M3 (GSTM3) is an important member of the GSTs that plays a critical role in the development of head and neck cancer (HNC). Several studies have investigated between the GSTM3 A/B polymorphism and risk of HNC, however, the results remain controversial. The aim of this meta-analysis is to evaluate the association between the GSTM3 A/B polymorphism and the risk of HNC.

**Methods:**

All eligible case-control studies published up to July 2013 were identified by searching PubMed and Web of Science. The HNC risk associated with the GSTM3 A/B polymorphism was estimated for each study by odds ratios (OR) together with its 95% confidence interval (CI), respectively.

**Results:**

Fourteen studies from ten publications with 2110 patients and 2259 controls were included. Overall, the GSTM3 A/B polymorphism was associated with a decreased risk of HNC using the dominant model, homozygote comparison model and heterozygote comparison model (OR = 0.82, 95%CI: 0.71–0.94; OR = 0.67, 95%CI: 0.49–0.94; and OR = 0.84, 95%CI: 0.73–0.97, respectively); besides, in stratification analyses by ethnicity, similar results were observed in Caucasian populations. Stratification by tumor site indicated that the GSTM3 polymorphism was associated with a decreased risk of laryngeal cancer under recessive model and homozygote comparison (OR = 0.52, 95%CI: 0.30–0.89; and OR = 0.50, 95%CI: 0.29–0.87, respectively); By stratifying source of control, decreased cancer risk was observed in hospital-based population under all genetic models (OR = 0.67, 95%CI: 0.56–0.81 for the dominant model; OR = 0.66, 95%CI: 0.46–0.95 for the recessive model; OR = 0.55, 95%CI: 0.37–0.83 for the homozygote comparison model, and OR = 0.70, 95%CI: 0.58–0.84 for the heterozygote comparison model).

**Conclusions:**

This meta-analysis suggests that the GSTM3 A/B polymorphism may be an important protective factor for HNC, especially of laryngeal cancer and Caucasian populations.

## Introduction

Head and neck cancer (HNC), including cancers of the oral cavity, pharynx, and larynx, is the sixth most common cancer worldwide [1]. HNC has been associated with high tobacco use and alcohol consumption [2]. There is also evidence that human papillomavirus (HPV) is responsible for HNC [3]. However, not all individuals who are smoking or drinking develop this group of fatal diseases in their normal life span, suggesting that individual genetic make-up may also be involved in disease aetiology.

Glutathione S-transferases (GSTs, Enzyme Commission 2.5.1.18) are a large family of phase II isoenzymes that catalyze the detoxification of reactive electrophilic compounds, including many environmental carcinogens (e.g., benzo[a]-pyrene and other polycyclic aromatic hydrocarbons) [4].

The GST genes are highly polymorphic and frequently inducible. Among the numerous GST genes (comprising types M1 to M5), the GSTM3 gene is located on chromosome 1q13.3 and has 2 alleles identified so far: GSTM3 A and GSTM3 B, of which the latter has a 3 bp deletion in intron 6, known as a recognition motif for the YY1 transcription factor [5]. GSTM3 B allele, having increased transcription potential, enhances detoxification activity of GSTM3-encoded protein [6]–[7]. This allele has also been linked to decreased risk of laryngeal carcinoma [8].

To date, GSTM3 A/B gene polymorphism has been extensively examined in association with risk of HNC [9]–[22]. However, the results have been inconclusive or inconsistent. So, we conduct a first meta-analysis to evaluate the association between GSTM3 A/B polymorphism and HNC susceptibility.

## Methods

### Search strategy

A literature research was conducted using PubMed and Web of Science up to July 2013 without language restrictions. Relevant studies were identified using the terms: [‘Glutathione S-transferase M3 or GSTM3’] AND [‘genetic polymorphism or polymorphisms or variant or SNP’] AND [‘head and neck cancer or HNC or oral cancer or pharyngeal cancer or laryngeal cancer or hypopharyngeal cancer or oropharyngeal cancer or nasopharyngeal cancer’]. The search was restricted to humans. Additional studies were identified by a hand search of references of original or review articles on this topic. If more than one geographic or ethnic heterogeneous group was reported in one report, each was extracted separately.

### Inclusion criteria and exclusion criteria

Studies were included if they met the following criteria: (1) studies that evaluated the association between the GSTM3 A/B polymorphism and HNC, (2) in a case-control study design, and (3) had detailed genotype frequency of cases and controls or could be calculated from the article text. While major exclusion criteria were: (1) case-only study, case reports, and review articles, (2) studies without the raw data of the GSTM3 A/B genotype, and (3) studies that compared the GSTM3 A/B variants in precancerous lesions and other cancers.

### Data extraction and quality assessment

Two investigators independently extracted data and reached consensus on all of the items. If they generated different results, they would check the data again and have a discussion to come to an agreement. If they could not reach an agreement, an expert was invited to the discussion. Data extracted from the selected articles included the first author's name, year of publication, country of origin, ethnicity, tumor site, genotyping methods, source of control, number of cases and controls. Tumor sites were categorized as oral, pharyngeal, laryngeal, and mixed HNC.

### Statistical analysis

Meta-analysis was performed using the Cochrane Collaboration RevMan 5.1 and STATA package version 12.0. The risk of HNC associated with the GSTM3 A/B polymorphism was estimated for each study by odds ratio (OR) and 95% confidence interval (95%CI). Four different ORs were calculated: the dominant model (AB+BB *vs.* AA), the recessive model (BB *vs.* AB+AA), heterozygote comparison (AB *vs.* AA), and homozygote comparison (BB *vs.* AA). A χ^2^-test-based Q statistic test was performed to assess the between-study heterogeneity [23]. We also quantified the effect of heterogeneity by *I* ^2^ test. When a significant Q test (*P*<0.05) or *I* ^2^>50% indicated heterogeneity across studies, the random effects model was used [24], or else the fixed effects model was used [25]. Before the effect estimation of GSTM3 A/B polymorphism in HNC, we tested whether genotype frequencies of controls were in HWE using χ^2^ test. We performed stratification analyses on ethnicity, tumor site and source of control. Analysis of sensitivity was performed to evaluate the stability of the results. Finally, potential publication bias was investigated using Begg' funnel plot and Egger's regression test [26]–[27]. *P*<0.05 was regarded as statistically significant.

## Results

### Study characteristics

**Figure 1:**
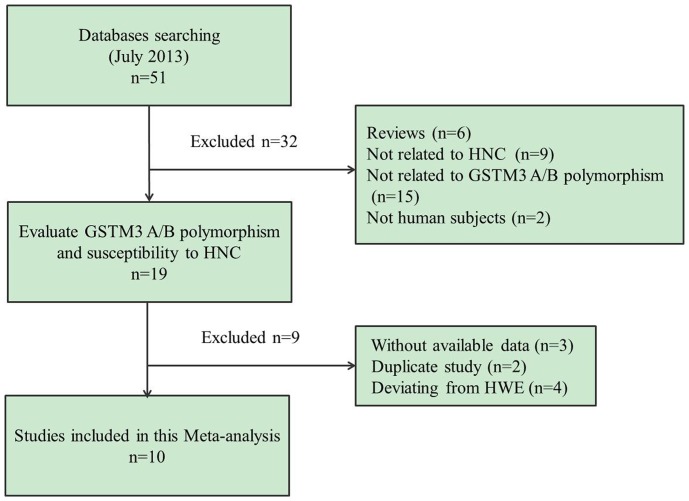
Flow chart showing study selection procedure.

The search strategy retrieved 51 potentially relevant studies. According to the inclusion criteria, 14 studies [9]–[22] with full-text were included in this meta-analysis and 37 studies were excluded. The flow chart of study selection in summarized in Figure 1. As shown in Table 1, because the studies [11], [13], [14] included two tumor types respectively and the study by Park et al [16] included two populations, we treated them separately in this meta-analysis. Furthermore, we removed 4 studies because their genotype distributions among the controls deviated from HWE [19]–[22]. Therefore, there were 14 case-control studies from 10 publications with 2110 cancer cases and 2259 controls concerning GSTM3 A/B polymorphism. Of the 14 eligible studies, two ethnicities were addressed: 13 studies on Caucasian populations and one study [16] on African-American. Four tumor sites were addressed: 6 studies focused on laryngeal cancer [9], [10], [12], [13], [14], [18], three studies on oral cancer [11], [16], 2 studies on pharyngeal cancer [11], [13], and 3 studies on mixed HNC [14], [15], [17].

**Table 1:** Characteristics of studies included in the meta-analysis.

					Genotyping	Source of	Age, mean±SD, year		Gender, n (male/female)		Genotype (case/control)				*P*_HWE_ 3
Study	Year	Country	Ethnicity	Tumor site	Methods	control	Case	Control	Case	Control	Total	AA	AB	BB	
Chatzimichalis [9]	2010	Greece	Caucasian	Laryngeal	TaqMan	PB^1^	66.5±8.54	62.7±9.62	77/11	76/26	88/102	81/92	7/10	0/0	0.60
Jahnke [10]	1996	England	Caucasian	Laryngeal	PCR	PB	61/58	NR^5^	246/23	NR	100/100	74/66	24/27	2/7	0.09
Jourenkova-Mironova [11]	1999	France	Caucasian	Oral	PCR-RFLP^4^	PB	54.4±10.2	54.9±11.1	113/8	163/9	67/172	49/125	16/42	2/5	0.52
Jourenkova-Mironova [11]	1999a	France	Caucasian	Pharyngeal	PCR-RFLP	PB	54.4±10.2	54.9±11.1	113/8	163/9	50/172	35/125	13/42	2/5	0.52
Jourenkova-Mironova [12]	1999b	France	Caucasian	Laryngeal	PCR-RFLP	PB	55.0	54.9	126/3	163/9	129/172	78/125	47/42	4/5	0.52
Matthias [13]	1998a	German	Caucasian	Pharyngeal	PCR-RFLP	HB^2^	NR	NR	NR	NR	118/173	89/116	22/47	7/10	0.09
Matthias [13]	1998a	German	Caucasian	Laryngeal	PCR-RFLP	HB	NR	NR	NR	NR	256/173	191/116	61/47	4/10	0.09
Matthias [14]	1998b	German	Caucasian	Oral/Pharyngeal	PCR-RFLP	HB	59.3±10	54.1±10.2	93/33	175/44	124/170	95/113	24/50	5/7	0.62
Matthias [14]	1998b	German	Caucasian	Laryngeal	PCR-RFLP	HB	62.0±10.3	54.1±10.2	249/23	175/44	262/170	200/113	58/50	4/7	0.62
Matthias [15]	2003	German	Caucasian	Head and neck	PCR-RFLP	HB	NR	NR	NR	NR	372/170	287/113	76/50	9/7	0.62
Park [16]	2000	USA	Caucasian	Oral	PCR-RFLP	HB	63.6(28–91)	60.4(28–91)	72/29	152/61	99/210	57/122	38/77	4/11	0.45
Park [16]	2000	USA	African-American	Oral	PCR-RFLP	HB	58.6(38–85)	59.6(34–88)	45/18	95/38	63/132	11/15	26/54	26/63	0.32
Rydzanicz [17]	2005	Poland	Caucasian	Head and neck	PCR-RFLP	PB	61.2±9.2	53.1±2.8	178/4	143/0	180/141	124/105	50/33	6/3	0.83
To-Figueras [18]	2002	Spain	Caucasian	Laryngeal	PCR-RFLP	PB	60±7	50±10	202/2	171/32	202/202	142/134	53/62	7/6	0.71

^1^ PB: population-based,

^2^ HB: hospital-based,

^3^ HWE, Hardy-Weinberg equilibrium; *P*_HWE_ was calculated by goodness-of fit χ^2^-test, *P*_HWE_<0.05 was considered statistically significant,

^4^ PCR-RFLP, polymerase chain reaction-restriction fragment length polymorphism.

^5^ NR, not reported.

### Quantitative data synthesis

**Figure 2:**
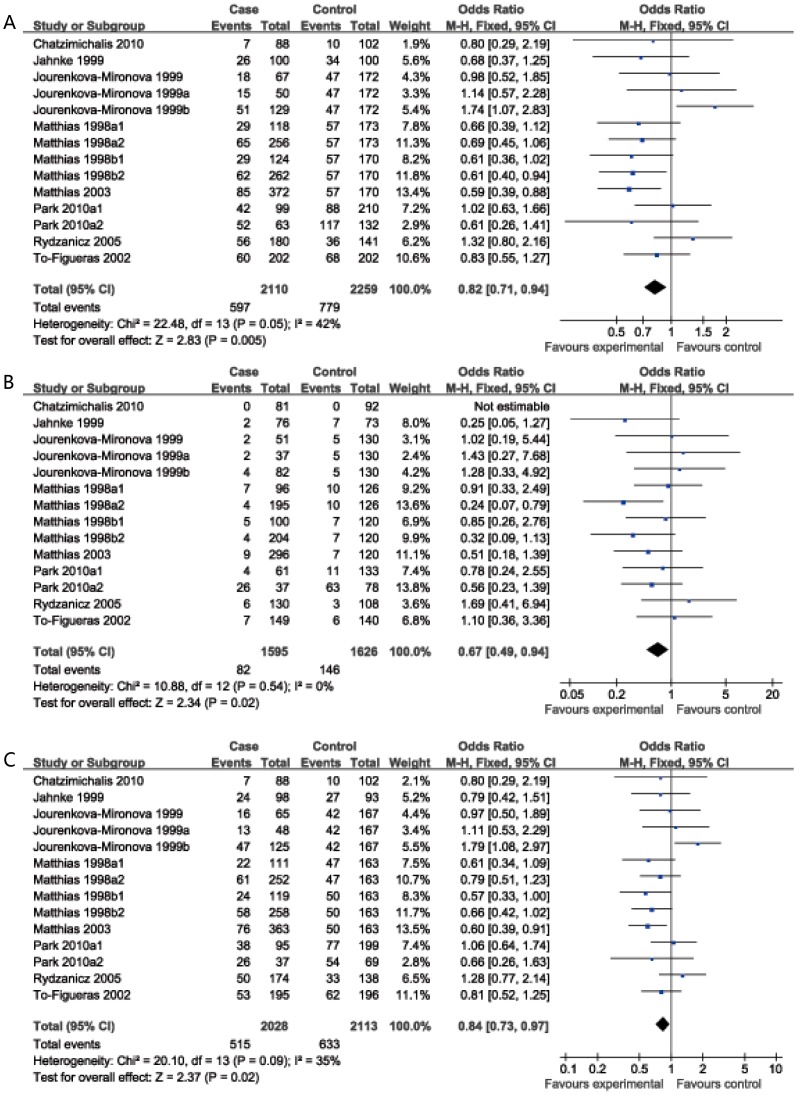
Forest plot of ORs for association between GSTM3 A/B and risk of HNC. (**A** dominant model; **B** BB *vs.* AA; **C** AB *vs.* AA).

As shown in Table 2, overall, the GSTM3 A/B polymorphism was associated with a decreased risk of HNC in three genetic models (OR = 0.82, 95%CI: 0.71–0.94 for the dominant model; OR = 0.67, 95%CI: 0.49–0.94 for the homozygote comparison model; OR = 0.84, 95%CI: 0.73–0.97 for heterozygote comparison model) (Figure 2), but no significant association was observed under the recessive model (OR = 0.74, 95%CI: 0.54–1.00)

**Table 2:** Stratified analysis of the GSTM3 polymorphism and HNC risk.

Variables	N^a^	AB+BB *vs.*AA			BB *vs.*AB+AA			BB *vs.*AA			AB *vs.*AA		
		OR^d^(95% CI^e^)	P^b^	I^2^	OR(95% CI)	P^b^	I^2^	OR(95% CI)	P^b^	I^2^	OR(95% CI)	P^b^	I^2^
**Total**	14	0.82(0.71,0.94)	0.05	42	0.74(0.54,1.00)	0.66	0	0.67(0.49,0.94)	0.54	0	0.84(0.73,0.97)	0.09	35
**Ethnicity**													
Caucasian	13	0.83(0.72,0.95)	0.04	45	0.73(0.51,1.03)	0.57	0	0.69(0.49,0.99)	0.47	0	0.85(0.73,0.98)	0.07	39
African-American	1	0.61(0.26,1.41)	N/A	N/A	0.77(0.42,1.41)	N/A	N/A	0.56(0.23,1.39)	N/A	N/A	0.66(0.26,1.63)	N/A	N/A
**Tumor site**													
Laryngeal	6	0.83(0.60,1.15)	0.04^c^	58	0.52(0.30,0.89)	0.24	27	0.50(0.29,0.87)	0.17	37	0.88(0.71,1.08)	0.08	50
Oral	3	0.92(0.65,1.31)	0.56	0	0.79(0.47,1.32)	0.95	0	0.69(0.35,1.34)	0.80	0	0.95(0.66,1.38)	0.67	0
Pharyngeal	2	0.81(0.53,1.22)	0.22	33	1.11(0.47,2.61)	0.76	0	1.02(0.43,2.42)	0.65	0	0.76(0.49,1.20)	0.21	37
Mixed HNC	3	0.77(0.46,1.29)	0.03^c^	72	0.88(0.45,1.72)	0.50	0	0.81(0.42,1.59)	0.39	0	0.76(0.46,1.25)	0.04^c^	68
**Source of control**													
PB	7	1.06(0.86,1.31)	0.21	29	0.96(0.55,1.69)	0.66	0	0.98(0.56,1.73)	0.60	0	1.07(0.86,1.34)	0.29	19
HB	7	0.67(0.56,0.81)	0.71	0	0.66(0.46,0.95)	0.56	0	0.55(0.37,0.83)	0.62	0	0.70(0.58,0.84)	0.64	0

^a^ Number of comparisons.

^b^ Test for heterogeneity.

^c^ Random-effects model was used when the P-value for heterogeneity test was <0.05, otherwise fixed-effects model was used.

^d^ OR, odds ratio.

^e^ CI, confidence interval.

In stratified analysis by ethnicity, we found that this polymorphism played different roles in Caucasian and African-American. In the Caucasian population, the GSTM3 A/B polymorphism had significant protective effects on the risk of HNC in three genetic models (OR = 0.83, 95%CI: 0.72–0.95 for the dominant model; OR = 0.69, 95%CI: 0.49–0.99 for the homozygote comparison model; OR = 0.85, 95%CI: 0.73–0.98 for heterozygote comparison model), but no significant association was observed under the recessive model (OR = 0.73, 95%CI: 0.51–1.03); while only one study focused on African-American, found that the GSTM3A/B polymorphism play an important role in risk for oral cancer (Table 2).

**Figure 3:**
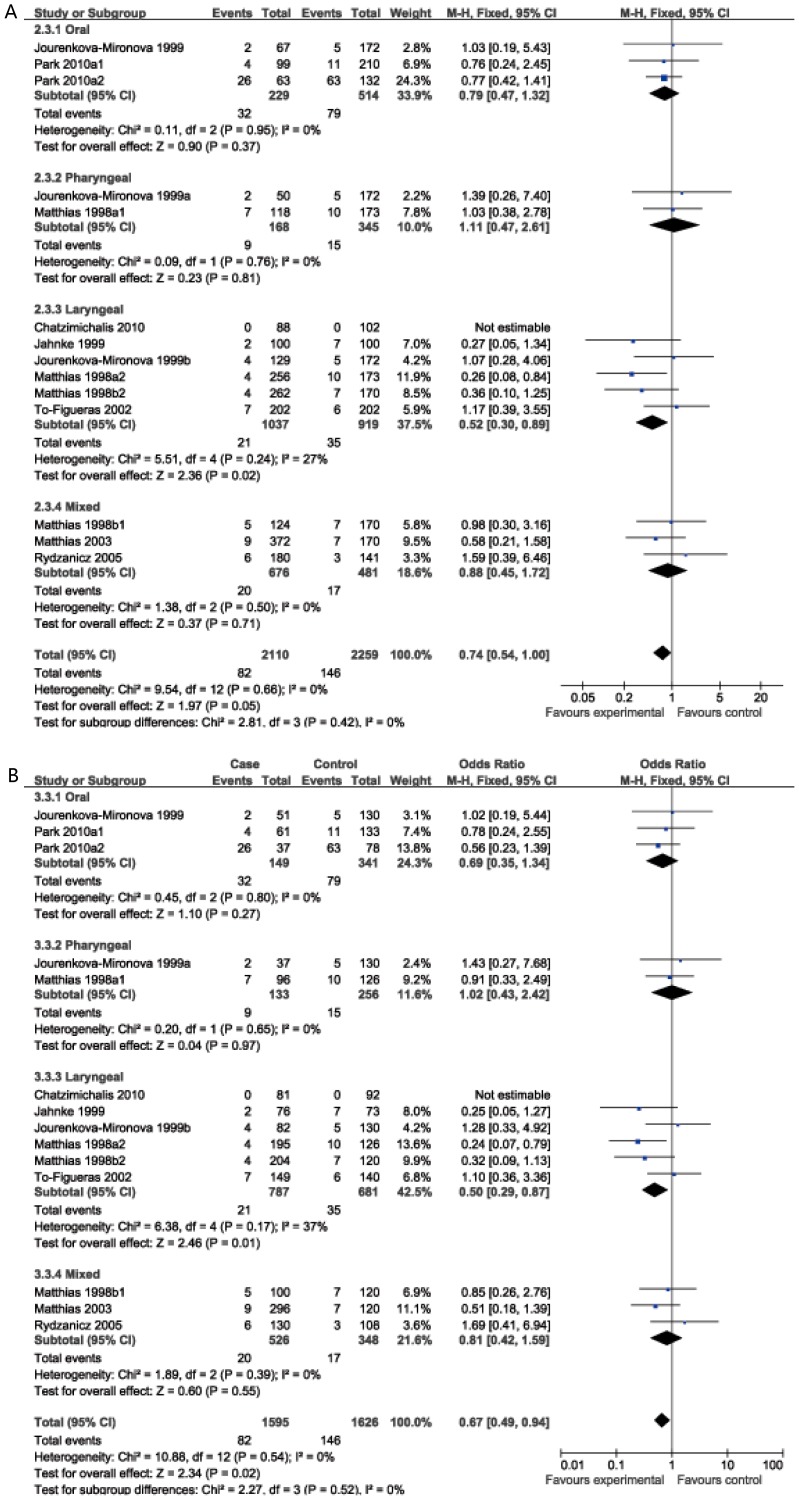
Subgroup analysis by tumor site of ORs with a fixed-effects model for association between GSTM3 polymorphism and HNC risk. (**A** recessive model; **B** BB *vs.* AA).

In the stratified analysis based on tumor site, significant associations were found in recessive and homozygote comparison model for laryngeal cancer (OR = 0.52, 95%CI: 0.30–0.89; OR = 0.50, 95%CI: 0.29–0.87 respectively); However, no significant association was found for either oral cancer or pharyngeal or mixed HNC (Table 2, Figure 3).

Stratification based on the source of controls showed significant associations between the GSTM3 A/B polymorphism and risk of HNC in the hospital-based subgroup (OR = 0.67, 95%CI: 0.56–0.81 for the dominant model; OR = 0.66, 95%CI: 0.46–0.95 for the recessive model; OR = 0.55, 95%CI: 0.37–0.83 for the homozygote comparison model, and OR = 0.70, 95%CI: 0.58–0.84 for the heterozygote comparison model). However, no significant association was found in the population-based subgroup (OR = 1.06, 95%CI: 0.86–1.31 for the dominant model; OR = 0.96, 95%CI: 0.55–1.69 for the recessive model; OR = 0.98, 95%CI: 0.56–1.73 for the homozygote comparison model; OR = 1.07, 95%CI: 0.86–1.34 for the heterozygote comparison model) (Table 2).

### Heterogeneity and sensitivity analysis

There were no inter-study heterogeneity among overall studies of the GSTM3 A/B polymorphism in all four genetic models (I^2^ = 42%, P_heterogeneity_ = 0.05 for the dominant model; I^2^ = 0%, P_heterogeneity_ = 0.66 for the recessive model; I^2^ = 0%, P_heterogeneity_ = 0.54 for the homozygote comparison model; I^2^ = 35%, P_heterogeneity_ = 0.09 for the heterozygote comparison model). Therefore, we used the fixed-effects model that generated wider CIs. However, it's worth noting that there was moderate heterogeneity among overall studies under dominant model, so we conducted stratified analysis by ethnicity, tumor cites and the source of the controls to find the potential sources of heterogeneity and we found that heterogeneity still exists in Caucasian population (I^2^ = 45%, P_heterogeneity_ = 0.04), laryngeal cancer (I^2^ = 58%, P_heterogeneity_ = 0.04) and mixed HNC (I^2^ = 72%, P_heterogeneity_ = 0.03), however, heterogeneity significantly reduced or removed among oral (I^2^ = 0%, P_heterogeneity_ = 0.56), pharyngeal cancer (I^2^ = 33%, P_heterogeneity_ = 0.22), population-based population (I^2^ = 29%, P_heterogeneity_ = 0.21) and hospital-based populations (I^2^ = 0%, P_heterogeneity_ = 0.71). Then, sensitivity analysis, after removing one study at a time, was performed to evaluate the stability of the results. We found that the estimated pooled odd ratio changed quite little, indicating that our results were statistically robust.

### Publication bias

**Figure 4:**
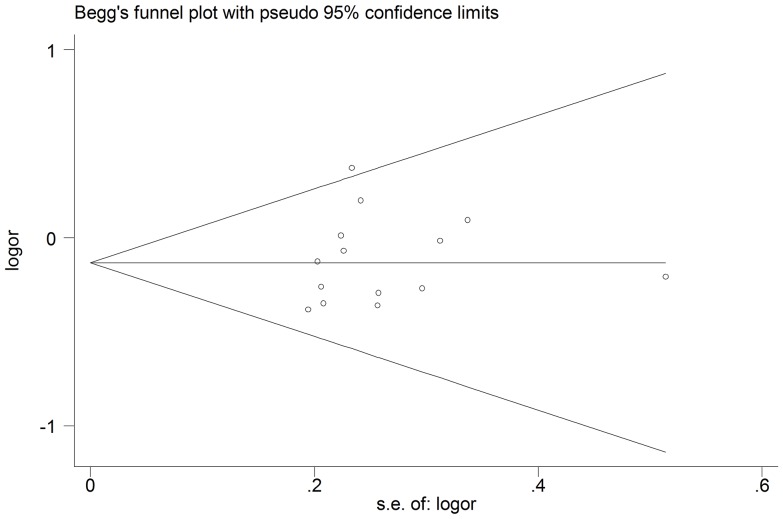
Begg's funnel plot for publication bias test. Each point represents an independent study for the indicated association under the dominant model.

Begg's funnel plot and Egger's test were performed to assess the potential publication bias in the available literature. The shape of funnel plots did not reveal any evidence of funnel plot asymmetry (Figure 4). Egger's test also showed that there was no statistical significance for the evaluation of publication bias (dominant model: *P* = 0.574, recessive model: *P* = 0.748, AB *vs.* AA: *P* = 0.718, BB *vs.* AA: *P* = 0.816).

## Discussion

To our knowledge, this is the first meta-analysis which comprehensively assessed the associations between GSTM3 A/B polymorphism and HNC risk. In this study, we found significant associations in the overall comparison using dominant model, homozygote comparison model and heterozygote comparison model. Individuals with the AB/BB genotype could have a decreased risk of HNC. Moreover, in the stratified analyses by several variables, including ethnicity, tumor site, and source of the controls, significant associations were observed in the Caucasian population, laryngeal cancer and hospital-based population.

GSTM3, a major member of the GSTs family, plays a role in metabolism of harmful agents, like polyaromatic hydrocarbons benzo(a)pyrene. The GSTM3 polymorphism could, therefore, confer different efficiencies in the metabolism of carcinogens and has been shown to modulate various cancers risk. Interestingly, it plays different roles in different cancers. Jain et al [28] reported patients who were heterozygous carriers of GSTM3 AB genotype had an enhanced risk for developing esophageal cancer. Loktionov et al [29] found that the GSTM3 B variant presence especially in combination with the GSTM1-null genotype is a risk factor for colorectal carcinogenesis, and Holley et al [30] suggested the GSTM3 AA genotype is associated with improved prognosis especially in those with GSTM1 null. While, some studies found that no significant association between GSTM3 polymorphism and lung [31], gallbladder [32] and adult brain tumor [33] risk was observed. For head and neck cancer, in a recent study, an increase in larynx cancer risk associated with GSTM3 AA genotype was suggested [14], similarly, Chatzimichalis et al [9] found that the presence of the GSTM3 B allele appears to be associated with a reduced risk of laryngeal SCC in a Greek population. Majumder et al [20] reported the GSTM3 AA genotype could increase the risk of oral leukoplakia and cancer among smokers; in contrast, Jourenkova-Mironova et al [11] found the GSTM3 AA genotype was not associated with oropharyngeal cancer risk, and Buch et al [19] reported that no association between the GSTM3 alleles and oral cancer risk was observed. These inconsistent results may be attributed to differences in genetic backgrounds, environmental factors, and other factors, such as small sample size or inadequate adjustment for confounding factors.

In this meta-analysis, we found that individuals with AB+BB genotype had a lower risk of developing HNC under dominant model, besides in the stratified analyses by ethnicity, tumor site, and source of control, we found that B allele carriers had a lower risk of HNC than A allele carriers in Caucasian population, laryngeal cancer and hospital-based population. The results may be explained that the B allele carriers could increase transcription of the GSTM3 gene and expression of GSTM3-related protein, then enhance detoxification activity. In addition, the pathways of carcinogen metabolism are complex, mediated by the activities of multiple genes (such as GSTM1, and CYP1A1) [34], [35]. GSTM3 is known to have overlapping substrate specificities with GSTM1 and GSTP1. And GSTM1/GSTP1 effects may be modulated by the GSTM3 genotype [36]. Individuals carrying variant genotypes on these loci might have higher levels of DNA adducts in the exposed tissues [37]. Consequently, there is a possibility that smokers carrying the GSTM1 or GSTP1 or GSTM3 risk genotype will be susceptible to cancer if the DNA adducts remain unrepaired. However, the specific role needs to be tested in future studies.

It would be hard to interpret results, if significant heterogeneity were present, in this meta-analysis, we did not find any obvious heterogeneity and publish bias across studies. While, it's worth noting that there was moderate heterogeneity among overall studies under dominant model, so stratified analysis by ethnicity, tumor cites and the source of the controls was performed and we found that moderate heterogeneity still exists in Caucasian population, laryngeal cancer and mixed HNC, however, heterogeneity significantly reduced or removed among oral, pharyngeal cancer, population-based population and hospital-based populations. The results above suggest that control selection and different tumor types may contribute to the moderate heterogeneity observed in the meta-analysis. Then sensitivity analyses were conducted by excluding one study successively, the estimated pooled OR changed quite little, strengthening the results from this meta-analysis.

Although considerable effort was made to test for the possible association between the GSTM3 A/B polymorphism and risk of HNC, some limitations of this meta-analysis should also be addressed. First, due to limited detailed data presented in the published studies, the potential effect of important risk factors to HNC was not examined, such as smoking, alcohol consumption and HPV status. Second, all studies are focused on Caucasian populations, which may generate selective bias. Third, our results were based on unadjusted estimates, without adjustment for age, gender, family history and other risk factors, while lacking of the information for the date analysis may cause serious confounding bias. Fourth, GSTM3 may influence susceptibility to HNC independently or with other genes such as GSTM1 and GSTP1. However, due to lack of individual data in the present review, we did not perform more detailed analyses, such as analyses of joint effects with other risk factors or gene-gene or gene-environment interactions.

In summary, this meta-analysis indicates that the GSTM3 A/B polymorphism may be an important protective factor for HNC, especially of laryngeal cancer and Caucasian populations. Further studies with standardized unbiased genotyping methods, homogeneous cancer patients, well-matched controls and multiethnic groups would be warranted.

## Supporting Information

Checklist S1 **PRISMA Checklist for this study.**
(DOC)

Checklist S2 **MOOSE Checklist for this study.**
(DOC)
