# Supplementary material for: GSTM3 A/B Polymorphism and Risk for Head and Neck Cancer: A Meta-Analysis
Source: PLoS One. 2014 Jan 8;9(1):e83851. doi: 10.1371/journal.pone.0083851 (PMC3885523; doi:10.1371/journal.pone.0083851)
Supplement: Checklist S2 — MOOSE Checklist for this study. (DOC) [file pone.0083851.s002.doc]

**MOOSE Checklist**

**GSTM3 A/B polymorphism and risk for head and neck cancer: a meta-analysis**

Yu Xu

Department of Otolaryngology, Renmin Hospital of Wu­han University, Wuhan, Hubei Province, China,

Jun Wang, Weiguo Dong

Department of Gastroenterology, Renmin Hospital of Wuhan University, Wuhan, Hubei Province, China.

Corresponding Author: Weiguo Dong

Department of Gastroenterology, Renmin Hospital of Wuhan University, 238 Jiefang Road, Wuhan 430060, Hubei Province, China.

E-mail: dwg@whu.edu.cn

Phone: 86-27-88041911

Fax: 86-27-88042292

1. **Reporting of background should include**

**Problem definition:** The GSTM3 A/B polymorphism and head and neck cancer risk

**Hypothesis statement:** GSTM3 A/B polymorphism contribute to decreasing head and neck cancer risk

**Description of study outcome:** The GSTM3 A/B polymorphism may be an important protective factor for HNC, especially of laryngeal cancer and Caucasian populations.

**Type of exposure or intervention used:** GSTM3 A/B polymorphism

**Type of study designs used:** Meta-analysis

**Study population:** Cancer patients and healthy controls

1. **Reporting of search strategy should include**

**Qualifications of searchers:** Xu Y and Wang J

**Search strategy, including time period include in the synthesis and keywords:**

PubMed from 1965 –July 2013

Web of Science from 1970 –July 2013

Glutathione S-transferase M3; polymorphism; head and neck cancer

**Effort to include all available studies, including contact with authors:** Yes

**Databases and registries searched:** PubMed, Web of Science

**Search software used, name and version, including special features used:** We did not employ any search software. EndNote was used to merge retrieved citations and eliminate duplications

**Use of hand searching:** Yes

**List of citations located and those excluded, including justification:** Figure 1 and Table 1

**Method of addressing articles published in languages other than English:** Translation software

**Method of handing abstracts and unpublished studies:** No unpublished studies were observed.

**Description of any contact with authors:** None

1. **Reporting of methods should include**

**Description of relevance or appropriateness of studies assembled for assessing the hypothesis to be tested:** Table 1

**Rationale for the selection and coding of data:** (1) studies that evaluated the association between the GSTM3 A/B polymorphism and head and neck cancer, (2) in a case-control study design, (3) had detailed genotype frequency of cases and controls or could be calculated from the article text.

**Documentation of how data were classified and coded:** Two investigators (Xu and Wang) used a standard protocol and data-collection form. They discussed with Pro. Dong, then decide the data.

**Assessment of confounding:** No restricted for the analysis. Conducted sensitivity analyses by eliminating the study that was not in HWE.

**Assessment of study quality, including binding of quality assessors; stratification or regression on possible predictors of study results:** The results of sensitivity analyses were very stable.

**Assessment of heterogeneity:** The chi-square-based *Q*-test and *I^2^* test

**Description of statistical methods in sufficient detail to be replicated:** A χ^2^-test-based Q statistic test was performed to assess the between-study heterogeneity. We also quantified the effect of heterogeneity by *I*^2^ test. When a significant Q test (*P*<0.05) or *I*^2^ >50% indicated heterogeneity across studies, the random effects model was used, or else the fixed effects model was used.

**Provision of appropriate tables and graphics:** We included the terms used for database search, 1 flow chart, 2 summary table, 2 forest plots of all studies,1 funnel plots to examine publish bias.

1. **Reporting of results should include**

**Graphic summarizing individual study estimates and overall estimate:** Table 2, Figure 2, Figure 3, Figure 4

**Table giving descriptive information for each study included:** Table 2

**Results of sensitivity testing:** Table 2

**Indication of statistical uncertainty of findings:** 95% confidence intervals were presented with all summary estimates, *P* values and results of sensitivity analyses.

1. **Reporting of discussion should include**

**Quantitative assessment of bias:** Sensitivity analyses indicate this non-significant association was stable.

**Justification for exclusion:** We excluded studies that had used different exposure or outcome assessment for the comparison groups, or no control group.

**Assessment of quality of included studies:** We discussed the results of the sensitivity analyses.

1. **Reporting of conclusions should include**

**Consideration of alternative explanations for observed results:** We discussed that potential unmeasured confounders such as differences of ethnicity, various cancer types, the source of controls, particular study, life style, environment background and other unknown factors may be the source of heterogeneity.

**Generalization of the conclusions:** The GSTM3 A/B polymorphism may be an important protective factor for HNC, especially of laryngeal cancer and Caucasian populations.

**Guidelines for future research:** Future studies should use standardized unbiased genotyping methods and homogeneous cancer patients and well-matched controls and include multiethnic groups.

**Disclosure of funding source:** No funding supported this study.
